# A randomized controlled trial of supervised remotely-delivered attention bias modification for posttraumatic stress disorder

**DOI:** 10.1017/S003329172200023X

**Published:** 2022-02-08

**Authors:** Yaron Alon, Omer Azriel, Daniel S. Pine, Yair Bar-Haim

**Affiliations:** 1 School of Psychological Sciences, Tel-Aviv University, Tel Aviv-Yafo, Israel; 2 Section on Developmental Affective Neuroscience, National Institute of Mental Health, Bethesda, MD, USA; 3 Sagol School of Neuroscience, Tel-Aviv University, Tel Aviv-Yafo, Israel

**Keywords:** Attention bias modification, posttraumatic stress disorder, randomized controlled trial, telemedicine

## Abstract

**Background.:**

Many individuals with posttraumatic stress disorder (PTSD) have limited access to first-line treatments, warranting the development of remotely-delivered treatments. Attention bias modification (ABM), targeting perturbed threat-related attentional patterns, shows promise when delivered in-person. However, previous studies found ABM to be ineffective when delivered remotely. Randomized clinical trials usually applied two variations of ABM: ABM away from threat or attention control training (ACT) balancing attention between threat-related and neutral stimuli. We tested remotely-delivered ACT/ABM with tighter supervision and video-based interactions that resemble in-clinic protocols. We expected to replicate the results of in-clinic trials, in which ACT outperformed ABM for PTSD.

**Methods.:**

In this double-blinded, parallel-group randomized controlled trial, 60 patients diagnosed with PTSD were randomized (ABM *n* = 30; ACT *n* = 30). Patients performed eight bi-weekly remotely-delivered supervised ABM/ACT sessions. Symptoms were assessed pre- and post-treatment with Clinician-Administered PTSD Scale 5 (CAPS-5) severity score and PTSD diagnosis as the primary outcomes. Current depressive episode, current anxiety-related comorbidity, and time elapsed since the trauma were examined as potential moderators of treatment outcome.

**Results.:**

Significant decrease in CAPS-5 severity scores and PTSD diagnosis was observed following both ACT and ABM with no between-group difference. Patients without depression or whose trauma occurred more recently had greater symptom reduction in the ACT than the ABM group.

**Conclusions.:**

Contrary to our expectation, symptoms decreased similarly following ACT and ABM. Moderator analyses suggest advantage for ACT in non-depressed patients and patients whose trauma occurred more recently. Further refinements in remotely-delivered ABM/ACT may be needed.

## Introduction

Posttraumatic stress disorder (PTSD) is associated with major functional impairments (American Psychiatric Association, 2013). Avoidance in PTSD often impedes treatment engagement (Hoge et al., 2004; Maguen et al., 2019), particularly for patients residing in remote areas (Bull, Krout, Rathbone-McCuan, & Shreffler, 2001). Even when patients do reach to clinics, first-line treatments for PTSD exhibit high attrition rates (Imel, Laska, Jakupcak, & Simpson, 2013; Maguen et al., 2019; Straud, Siev, Messer, & Zalta, 2019). This highlights the need for better-tolerated, home-delivered treatments (Morland et al., 2020). In this proof-of-concept study, we tested the acceptability, feasibility, and efficacy of one such potential treatment, attention bias modification (ABM), delivered remotely at patients’ homes.

ABM targets aberrant attentional patterns in psychopathology. In PTSD, two types of attentional biases have been identified: (a) attention bias toward threats (Fani et al., 2012; Lazarov et al., 2019*b*); and (b) threat-related attention bias variability (ABV), reflected in elevated dynamic fluctuations between threat vigilance and threat avoidance over time (Alon, Naim, Pine, Bliese, & Bar-Haim, 2019; Iacoviello et al., 2014; Naim et al., 2015). Accordingly, in-clinic randomized controlled trials (RCTs) for PTSD typically contrasted ABM designed to shift attention away from threats and attention control training (ACT) designed to reduce ABV (Badura-Brack et al., 2015; Lazarov et al., 2019*a*). Results suggest that both induce symptom reduction (Badura-Brack et al., 2015; Lazarov et al., 2019*a*; Schoorl, Putman, & van der Does, 2013). However, recent reports suggest an advantage for ACT over ABM in PTSD symptom reduction (Badura-Brack et al., 2015; Lazarov et al., 2019*a*). Thus, ABV may more closely relate to PTSD symptoms (Badura-Brack et al., 2015).

Unlike in-clinic ABM, remotely-delivered ABM generally fails to show advantage for ACT/ABM in symptom reduction (for reviews see: Linetzky, Pergamin-Hight, Pine, & Bar-Haim, 2015; Mogoaşe, David, & Koster, 2014). This could reflect several factors. First, insufficient performance monitoring at home can reduce adherence. Second, clinic attendance may promote behavioral activation or bias samples to patients with mild avoidance symptoms. Third, physical conditions at home might limit efficacy through effects of distractions or create variability in the delivered treatments.

To address these issues, we developed a supervised remotely-delivered ABM protocol that resembles typical in-clinic protocols: (a) Treatment sessions were scheduled in advance; (b) patients were accompanied during their pre-scheduled training sessions using video conferencing; (c) the physical environment for training was adapted before and during the session; (d) proper task parameters were remotely verified. Specifically, before each session, the experimenter and the patient verified that environmental noise was minimal, that no one was expected to enter the room during the session, and that the patient was seated comfortably in front of a desk on which the computer screen was placed.

In this proof-of-concept study, we examined the feasibility, acceptability, and efficacy of this supervised ABM protocol, expecting to replicate in-clinic results showing that ACT outperforms ABM. Three potential moderators of efficacy, implicated in previous research, were explored: comorbid depression, comorbid anxiety disorders, and time elapsed since trauma.

PTSD is highly comorbid with major depression (Hankin, Spiro, Miller, & Kazis, 1999; Norris, Murphy, Baker, & Perilla, 2004; Spinhoven, Penninx, van Hemert, de Rooij, & Elzinga, 2014). Individuals with depression tend to have a general motor deceleration (Bennabi, Vandel, Papaxanthis, Pozzo, & Haffen, 2013) and deficits in executive functions (McDermott & Ebmeier, 2009). These deficits might restrict benefits from reaction-time-based interventions that typically use short presentation durations (Armstrong & Olatunji, 2012; De Raedt & Koster, 2010). Therefore, patients with PTSD and comorbid depression might benefit less from the current interventions than patients without depression.

Previous studies found ABM to outperform ACT for anxiety disorders (Mogoaşe et al., 2014), whereas the opposite pattern has been found in PTSD (Badura-Brack et al., 2015; Lazarov et al., 2019*a*). Thus, comorbid anxiety disorders may moderate ABM/ACT efficacy for PTSD.

Finally, time elapsed since trauma may moderate outcomes (Segal, Pine, & Bar-Haim, 2020). Recently acquired maladaptive threat-related attentional patterns may be less entrenched than chronic patterns.

We expected that both treatments would show high acceptability expressed in low attrition rates and high engagement in treatment. We expected ACT to outperform ABM away from threat in clinical efficacy [greater reduction in Clinician-Administered PTSD Scale 5 (CAPS-5) severity scores and PTSD diagnosis, our pre-registered primary outcomes]. We also expected ACT to reduce ABV and ABM to reduce attention bias toward threat, replicating the results of previous in-clinic RCTs (Badura-Brack et al., 2015; Lazarov et al., 2019*a*). Finally, we expected that the clinical effects would be greater among individuals without comorbid depression or anxiety disorders, and among patients for whom shorter times had elapsed since trauma.

## Method

### Participants

**Fig. 1.**
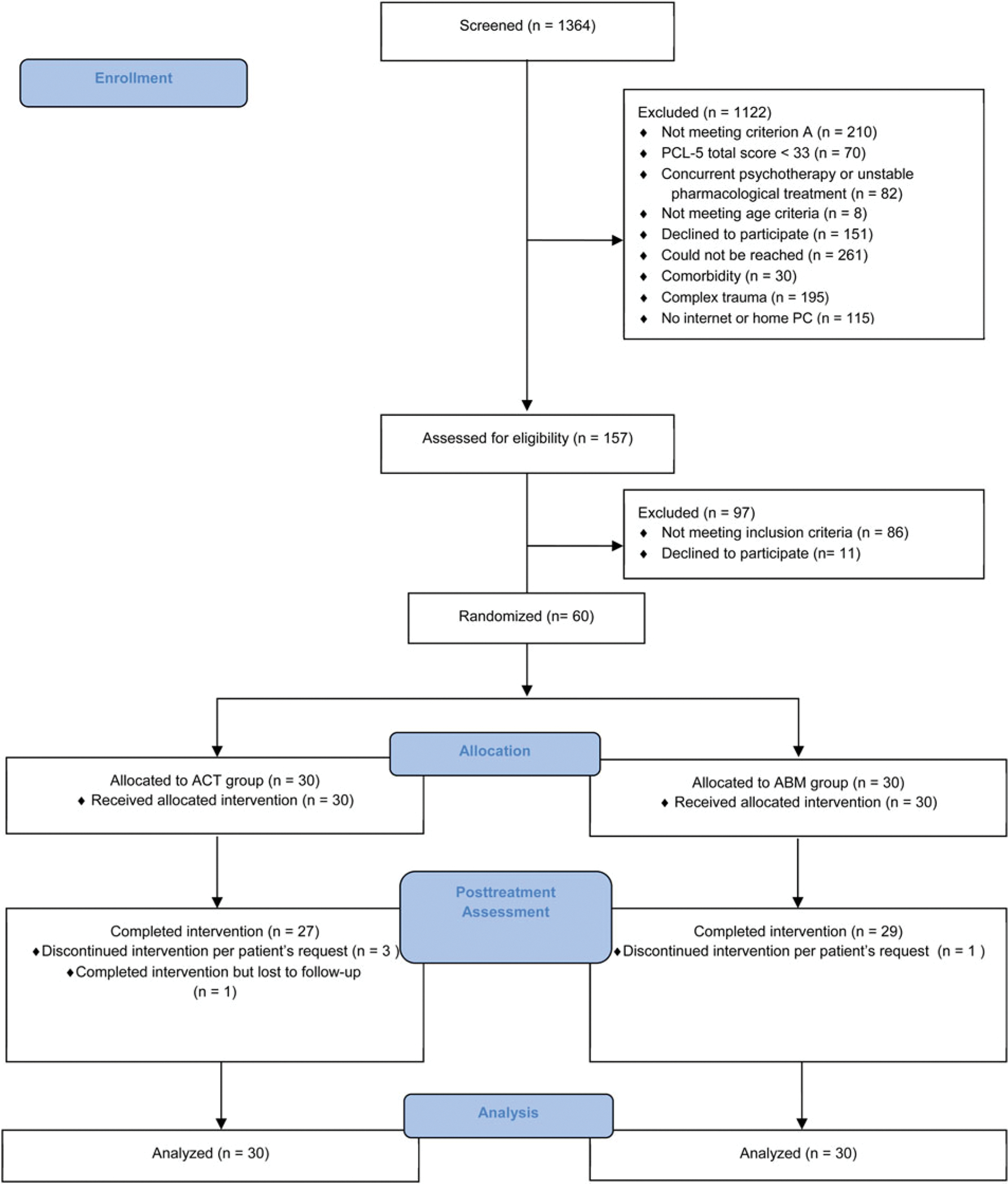
CONSORT flow diagram.

A CONSORT diagram appears in Fig. 1. Participants were recruited via social media inviting individuals distressed by a traumatic event to receive treatment as part of research. Advertisement targeted regions where accessibility to psychological treatment is limited. Potential participants were telephone-screened for PTSD symptoms using the PTSD Checklist-5 (PCL-5; Blevins, Weathers, Davis, Witte, & Domino, 2015). Those reporting a traumatic event (DSM-5 criterion A), having probable PTSD (PCL-5 score ⩾33), and affirming access to a computer, Internet, and videoconferencing at home were invited for an online clinical assessment. Out of 1364 applicants, 157 were remotely assessed by a clinician; of those, 86 did not meet inclusion criteria and 11 eligible individuals declined participation. Sixty patients with PTSD were enrolled (*M*_age_ = 39.68 years, s.d. = 12.19, range = 21–65, 40 females).

Inclusion criteria: (a) a DSM-5 PTSD diagnosis ascertained via CAPS-5 interview; (b) age 18–65 years; (c) access to a computer, Internet, web cam, microphone, and speakers at home. Exclusion criteria: (a) current or past psychosis, bipolar disorder, manic or hypomanic episode; (b) epilepsy or brain injury; (c) suicidal ideation; (d) drugs/alcohol abuse; (e) pharmacological treatment not stabilized for at least 3 months or concurrent psychotherapy. A stable pharmacological treatment did not lead to exclusion if it did not change during the study period. Some participants reported stable pharmacological treatment initiated at least 3 months prior to the study (seven in ACT, six in ABM). No changes in pharmacological treatment were reported during the trial.

Trauma experiences indexed via the CAPS-5 interview and comorbidities assessed by the Mini-International Neuropsychiatric Interview (Sheehan et al., 1998) are reported in Table 1. Thirty participants (50%) reported that they had experienced additional traumas to the main trauma encoded in the CAPS-5 interview.

The study was approved by the Tel Aviv University Ethics Committee. Informed consent was provided online. ClinicalTrials.gov identifier: NCT04228133.

### Diagnostic and self-report measures

#### Primary outcome

The CAPS-5 (Weathers et al., 2018) is a semi-structured interview probing symptoms of PTSD according to DSM-5. Two measures were derived: (1) total severity score; and (2) PTSD diagnosis (present/absent).^1^ CAPS-5 also afforded assessment of time elapsed since the traumatic event. Interviews were conducted by three independent graduate-level clinical psychologists in internship, trained to 85% reliability with an experienced clinical psychologist. The clinicians were blind to group assignment. Cronbach’s αs in the current sample were 0.71 and 0.88 at pre- and post-treatment, respectively.

#### Secondary outcome

The PTSD checklist for DSM-5 (PCL-5; Blevins et al., 2015) is a self-report questionnaire reviewing symptoms of PTSD according to DSM-5. Cronbach’s αs for the total severity score in the current sample were 0.79 and 0.93 at pre- and post-treatment, respectively.

#### Additional clinical measures

The Patient Health Questionnaire 9 (PHQ-9; Kroenke, Spitzer, & Williams, 2001) is a self-report measure assessing depression symptoms according to DSM-IV. Cronbach’s *α*s for the total severity score in the current sample were 0.78 and 0.87 at pre- and post-treatment, respectively.

The Clinical Global Impression Severity (CGI-S) and Improvement (CGI-I) scales (Guy, 1976) are single items, assessing global severity and improvement of illness, respectively, using seven-point scales. CGI-S and CGI-I were scored by the independent evaluators. The CGI-S was scored at pre- and post-treatment, CGI-I was scored at post-treatment referring to the change in clinical condition over time. The CGI-S/I have good sensitivity to clinical change (Berk et al., 2008).

The Credibility/Expectancy Questionnaire (CEQ; Devilly & Borkovec, 2000) is a scale assessing expectancy of clinical improvement and perceived treatment credibility. The CEQ was administered at pre-treatment, after an explanation of the study’s rationale and procedures and before randomization and reflects pre-treatment expectancies. Cronbach’s *α* in the current sample was 0.84.

The Mini-International Neuropsychiatric Interview (Sheehan et al., 1998) is a structured interview for DSM-IV and ICD-10 diagnoses, used here to assess baseline comorbidities.

### Attention bias and attention bias variability

Attention bias and ABV were measured using a faces-based dot-probe task (the Tel Aviv University and National Institute of Mental Health ABMT Initiative; http://people.socsci.tau.ac.il/mu/anxietytrauma/research/). In each trial, a fixation cross appeared (500 ms), and replaced by a pair of faces, one with a neutral expression, the other with an angry expression (500 ms). Then, an arrowhead pointing right (‘>‘) or left (‘<‘) appeared at the location of one of the faces until response. Participants were requested to respond as quickly and accurately as possible to the direction of the arrowhead. The arrowheads appeared at the locations of the neutral and angry faces with equal probability. In total, 160 trials were presented. Incorrect trials, trials faster than 200 ms or slower than 2000 ms, and trials deviating more than 2.5 s.d.s from the patient’s mean were removed (7.1% of trials). Attention bias was calculated as the difference between the mean RT of trials in which the arrowhead appeared at the neutral face location and the mean RT of trials in which the arrowhead appeared at the angry face location. Split-half reliability in the current sample was 0.03 and 0.31 at pre- and post-treatment, respectively. ABV was calculated as per Alon et al. (2019). Briefly, the standard deviation of attention bias scores calculated using a moving average was divided by participant’s mean RT. Split-half reliabilities were 0.22 and 0.21 at pre- and post-treatment, respectively.

### Treatment conditions

Both treatment conditions used the dot-probe task described above with different face stimuli than those applied in the attention bias and ABV measurement task (160 trials per session).

#### Attention control training

The ACT protocol was designed to balance participants’ attention between threat and neutral stimuli to reduce ABV. In this condition, the targets appeared with equal probability at the neutral and angry face locations.

#### Attention bias modification away from threat

The ABM condition is the same as the ACT condition with one exception – the targets always appeared at the neutral face location to induce attentional shift away from threats.

### Procedure

Study design was a double-blind parallel-group RCT: two groups (ACT and ABM) and two assessment time points (pre- and post-treatment), such that the independent clinicians, personnel staff, and participants were blind before and during treatment to group allocation, which was coded with a random number for each condition. Participants were randomly assigned to conditions in a 1:1 ratio using a list created with a random number generator before enrollment started. Group assignment was monitored by a staff member not involved in the study in any other capacity.

Potential participants were informed about the study’s rationale and procedures and those interested provided an online informed consent. Participants then completed the self-report questionnaires using Qualtrics surveys (http://www.qulatrics.com) and the structured diagnostic interviews, using the Google Meet App (https://apps.google.com/meet/). Those who met study criteria were randomized to ACT or ABM and completed eight bi-weekly sessions over 4 weeks at home using an app remotely installed on their local hard drive. A text reminder was sent to patients a day prior to each session. Each session started with a video conference. The assistant presented him/herself and guided the patient to enter the app. The assistant verified that the patient was sitting comfortably in front of the screen and that potential distractions were minimized (e.g. closing windows and doors to minimize noise, putting aside the smartphone, verifying no one is expected to enter the room). The patient then began the training session during which the assistant remotely monitored the conditions in the room. At this stage, the assistant’s own face and voice streaming was shut. If a minor interruption occurred (e.g. an incoming text message that induced a notification sound but not answered), participants were asked at the end of the session to reduce such distractions in the upcoming sessions; if there was a major interruption (e.g. the patient started talking to a household member during training), the assistants turned on their microphone and asked to cease the interruption assisting the patient to redirect to the training. At the end of the session, the assistant streaming was reinstated, the session’s data were stored, and the session was ended with a reminder of the next session. Assistants were instructed not to answer questions during training and not to develop prolonged discussions of any kind with the patients. In sessions 1 and 8, participants also completed the dot-probe measurement task before and after training, respectively. Post-treatment clinical assessment took place 1–2 weeks after the last treatment session. Following the last assessment, participants provided free feedback on their experience during treatment. The study was conducted between January 2020 and April 2021 until 60 participants were recruited as planned. Participants were administered with the treatment at their homes. Assistants and clinicians were located at Tel Aviv University.

### Data analysis

To explore group differences on baseline descriptive statistics, independent samples *t* tests and χ^2^ were computed. Clinical effects were analyzed using the intent-to-treat principle computing random-effect time-series models in generalized estimating equations (GEE; Liang & Zeger, 1986; Zeger, Liang, & Albert, 1988). GEE considers correlations between repeated measurements while addressing missing data through estimated marginal means based on the entire sample of all randomized participants. Wald’s χ^2^ (Rotnitzky & Jewell, 1990) was calculated to test whether the coefficient of the predictors in the models were significantly different from zero. The GEE models tested time (pre-treatment, post-treatment) by group (ACT, ABM) effects on PTSD primary (CAPS-5) and secondary (PCL-5) outcome measures, depression (PHQ-9), clinical global severity (CGI-S), and attentional bias measures (threat-related attention bias and ABV). A χ^2^ test examined PTSD diagnosis according to CAPS-5 (present/absent) post-treatment.

To evaluate expectancy and treatment credibility effects on clinical improvement, Pearson correlations were calculated between pre-treatment expectancy and credibility scores and pre- to post-treatment change in the clinical outcomes (CAPS-5, PCL-5, PHQ-9).

Additional exploratory analyses examined the moderating role of comorbid depression or anxiety disorders (social phobia, generalized anxiety disorder, panic disorder, or agoraphobia) on PTSD symptom reduction indexed by the interaction effect of comorbid current depressive episode/anxiety-related disorder (present/absent) by group (ACT, ABM) and by time (pre-treatment, post-treatment). To consider time since trauma (indexed as the main trauma in CAPS-5) on the relation between group (ACT, ABM) and PTSD symptom change over time (the difference rate in CAPS-5 total score from pre- to post-treatment), hierarchical multiple regression analysis was conducted using PROCESS macro in SPSS (model 1) (Hayes & Rockwood, 2017). Statistical tests were two-sided, *α* ⩽ 0.05.

Pre-treatment dot-probe data were missing for one patient in the ABM group due to a technical failure; the data of the CAPS-5, CGI-S, and CGI-I from post-treatment were missing for five patients (four in ACT, one in ABM). Of these five participants, two agreed to complete the self-report questionnaires online (i.e. PCL-5 and PHQ-9) but declined the clinical interview (two in ACT).

Power analyses were conducted using G*Power3.1.9.2 software (Faul, Erdfelder, Lang, & Buchner, 2007). Based on previous studies comparing the efficacy of ACT and ABM for PTSD (Badura-Brack et al., 2015; Lazarov et al., 2019*a*), we wanted to allow the detection of medium effect size of 0.65 with CAPS-5 severity score as the dependent variable, at 0.80 power and *α* = 0.05, which would require 58 participants. We decided to enroll 60 participants.

## Results

Baseline characteristics are described in Table 1. No group differences were noted in any of these variables between the two treatment groups at baseline, all *ps* > 0.06.

### Treatment adherence

Fifty-six participants (93.3%) completed all the sessions: 90% in ACT and 96.67% in ABM, χ^2^ = 0.3, *p* = 0.62. The remaining four participants (6.7%) completed four sessions on average (50% of all treatment sessions) before they requested to withdraw from treatment. Mean accuracy on the dot-probe task was 97.39%. In 100% of the sessions, participants completed all 160 task trials. No adverse events were reported during the trial.

### Feasibility

On 10.5% of all sessions, technical problems led to the initiation of a standard phone call or a WhatsApp video call instead of Google Meet. On 12.3% of all sessions, interruptions that may have distracted participants’ attention from the task were noted as follows: background noise from the street or inside the house (6.5%), someone entering the room during training (3%), patient’s phone was ringing/vibrating (2.2%), patient lit up a cigarette (0.6%). Entering interruptions as a covariate to analyses did not change the below reported results. Patients’ free feedback post-treatment about issues warranting improvement and merits of the novel protocol is described in online Supplementary Table S1.

### Clinical outcomes

#### Primary outcomes: PTSD symptom severity and PTSD diagnosis post-treatment (CAPS-5)

GEE analysis of CAPS-5 severity scores revealed an overall decrease in clinician-rated PTSD severity from pre- to post-treatment, Wald χ^2^ = 27.33, *p* < 0.001, *d* = 0.68. The time-by-group interaction was not significant, Wald χ^2^ = 0.067, *p* = 0.796. PTSD diagnosis rates according to CAPS-5 at the end of the treatment did not differ between the two conditions (ACT: 50%, ABM: 55.2%), χ^2^ = 0.147, *p* = 0.701.

#### Secondary outcome: self-reported PTSD symptoms (PCL-5)

GEE analysis of the PCL-5 indicated a reduction in self-reported PTSD severity from pre- to post-treatment, Wald χ^2^ = 62.88, *p* < 0.001, *d* = 1.20. The time-by-group interaction was not significant, Wald χ^2^ = 0.164, *p* = 0.686.

#### Additional clinical measures

GEE analyses indicated a significant symptom reduction over time in both depression (PHQ-9), Wald χ^2^ = 34.82, *p* < 0.001, *d* = 0.83, and in clinical global severity (CGI-S), Wald χ^2^ = 28.07, p < 0.001, *d* = 0.76. Here too, the time-by-group interactions were not significant: Wald χ^2^ = 0.02, *p* = 0.886 and Wald χ^2^ = 1.184, *p* = 0.277, respectively. An independent sample *t* test of clinical global improvement (CGI-I) revealed no significant difference between the ACT and ABM conditions, *t*(53) = 0.381, *p* = 0.705.

#### Change in attention bias and ABV from pre- to post-treatment

ABV and attention bias were not correlated to baseline CAPS-5 or PCL-5 scores, all *r*s < 0.19, *p*s > 0.153. GEE analysis indicated that ABV decreased from pre- to post-treatment in both ACT and ABM, Wald χ^2^ = 20.44, *p* < 0.001*, d* = 0.91. The time-by-group interaction was not significant, Wald χ^2^ = 0.90, *p* = 0.343. Attention bias did not change with treatment, either as a main effect, Wald χ^2^ = 0.062, *p* = 0.804, or in interaction with group, Wald χ^2^ = 0.173, *p* = 0.677.

#### Effects of expectancy and treatment credibility on clinical outcome

Patients found the treatment rationale moderately credible (*M* = 5.77, s.d. = 1.78) and expected a mean of ∼52% improvement in symptoms. Expectancy and credibility were not associated with change in CAPS-5, PCL-5, and PHQ-9 scores, *r*s < 0.21*, p*s > 0.102.

#### Moderators of treatment outcome

**Fig. 2.**
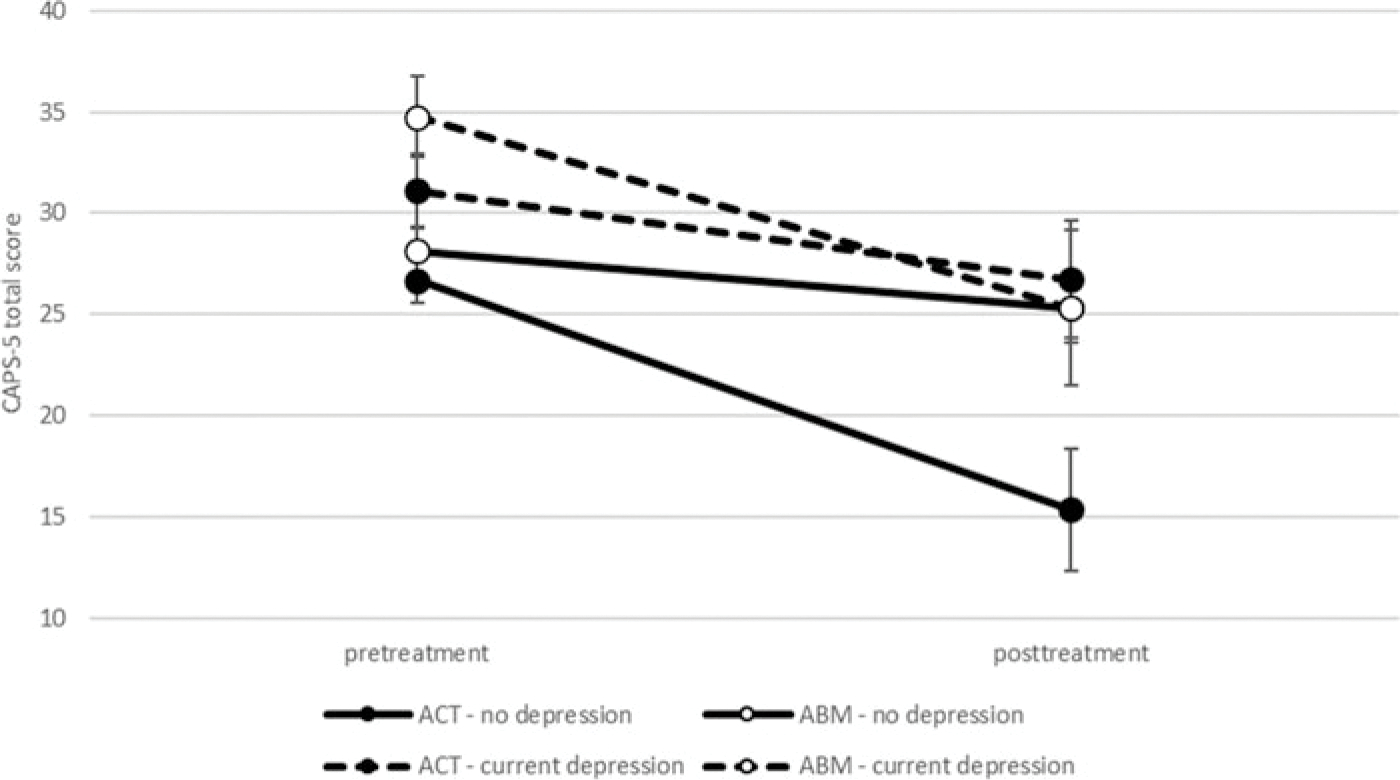
Clinician-rated PTSD symptom severity (CAPS-5) as a function of current severe depressive episode, condition, and time.

Current depressive episode: GEE analysis revealed a three-way interaction between current depressive episode (present/absent), time (pre-treatment/post-treatment), and group (ACT/ABM), Wald χ^2^ = 8.52, *p* = 0.036, *d* = 0.55 (Fig. 2). Further analyses indicated a significant time-by-group interaction only among the non-depressed patients, Wald χ^2^ = 6.39, *p* = 0.011, but not among patients with co-morbid depression, Wald χ^2^ = 1.84, *p* = 0.175, indicating greater symptom reduction in non-depressed ACT *v*. ABM.

##### Comorbid anxiety disorder:

GEE analysis did not yield a significant three-way interaction between comorbid anxiety disorder (present/absent), time (pre-treatment/post-treatment), and group (ACT/ABM), Wald χ^2^ = 2.07, *p* = 0.724.

##### Time elapsed since trauma:

**Fig. 3.**
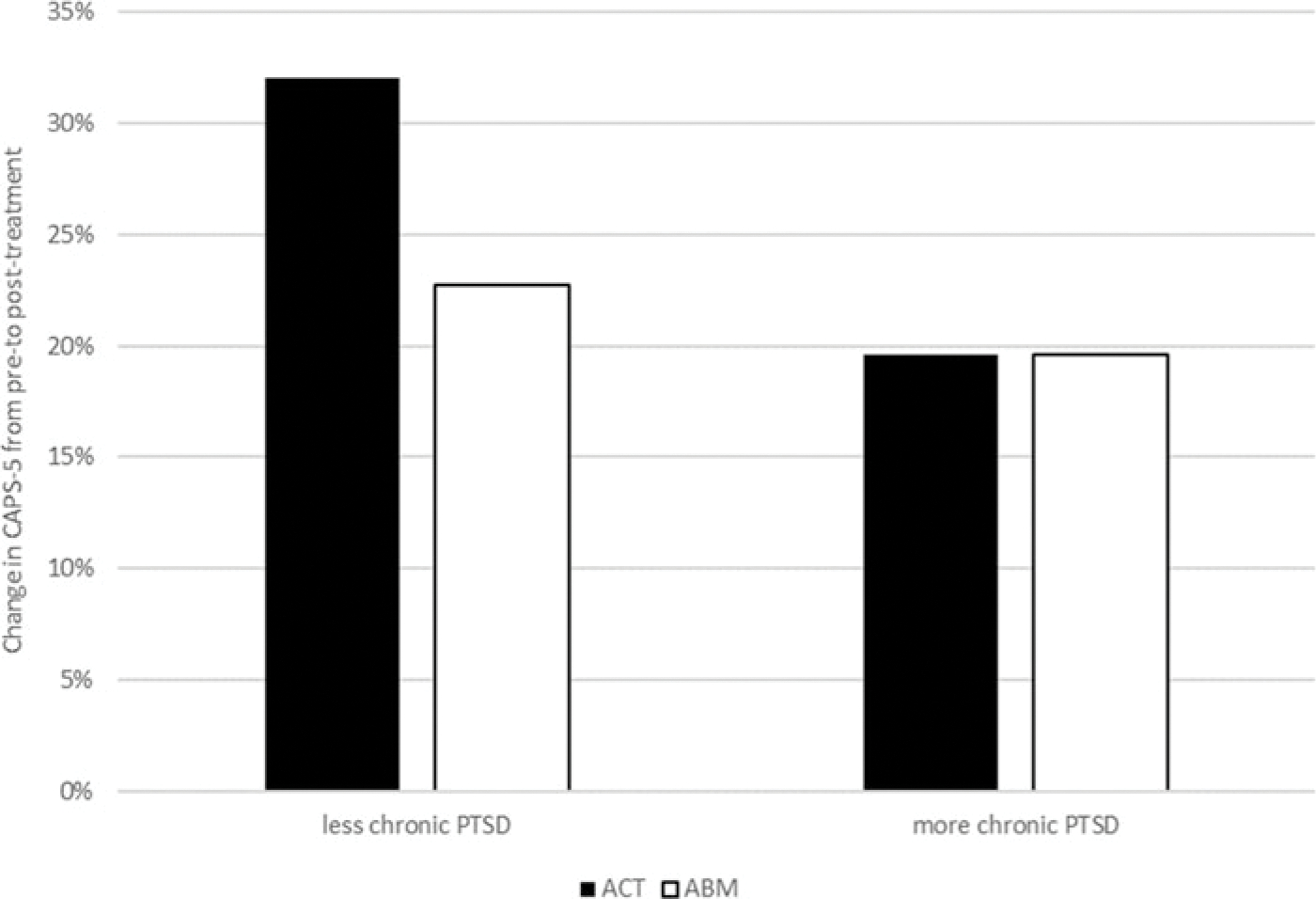
Illustration of change in clinician-rated PTSD symptom severity (CAPS-5) as a function of condition and PTSD chronicity. The graph illustrates decomposition of the interaction between time since trauma and treatment condition based on splitting the time since trauma variable into those below or above the average.

The hierarchical multiple regression on change in clinician-rated PTSD severity from pre- to post-treatment revealed that time since trauma was associated with PTSD symptoms change, *b* = −0.03, S.E. = 0.01, *β* = −1.04, *p* = 0.026, and this effect was qualified by a significant group-by-time-since-trauma interaction, *F*_(1,51)_ = 4.16, *p* = 0.046, *R*^2^ *change* = 0.07. Figure 3 illustrates decomposition of this interaction splitting the time since trauma variable into those below or above the average. ACT was associated with greater symptom reduction compared to ABM among patients who had experienced the trauma more recently. This effect is not apparent in patients whose trauma occurred longer ago.

## Discussion

This study tested the acceptability, feasibility, and efficacy of supervised remotely-delivered ABM and ACT for patients with PTSD. The remote treatment was designed to address problems in previous remotely-delivered protocols for anxiety disorders (Linetzky et al., 2015) and PTSD (Niles et al., 2020). Results indicate good adherence with 6.7% dropout, which is lower than the typically reported dropout in other PTSD treatments (Imel et al., 2013; Maguen et al., 2019; Straud et al., 2019) and Internet-delivered CBT (Lewis, Roberts, Simon, Bethell, & Bisson, 2019). In addition, treatment completers completed 100% of treatment task trials with a very high accuracy rate, indicating good acceptability. However, 8.4% of all screened individuals who contacted the study could not participate and receive treatment due to lack of a home PC or Internet connection. In addition, although we attempted to minimize distractions during the remote treatment, 12.3% of the treatment sessions were interrupted and on 10.5% of the sessions video conferencing was substituted by communication over the participants’ smart phones rather than the intended application. This suggests that control over patients’ home environment was still limited compared to the control in-clinic.

Contrary to our expectations (Badura-Brack et al., 2015; Lazarov et al., 2019*a*), even though symptoms lessened with a medium-to-large effect size, reductions did not differ between groups. Instead, results align with studies showing no group differences (Schoorl et al., 2013; Segal et al., 2020). The current RCT was similar to previous RCTs in design (e.g. Badura-Brack et al., 2015; Lazarov et al., 2019*a*), but also differed on some aspects that may have affected the results. First, the target population in Badura-Brack et al. (2015) was male veteran patients, whereas here the patients were both males and females who have experienced diverse traumatic events. It could be that conclusions drawn on the efficacy of ACT/ABM for PTSD in previous RCTs on male veterans do not generalize to other PTSD populations. Future studies could investigate this possibility directly with larger samples. The current study also differed from Badura-Brack et al. (2015, Study 1), which applied word stimuli rather than facial stimuli. Lastly, the current study differed from Lazarov *et al*. (2019*a*), where the ABM condition could be either away or toward threat depending on the patient’s baseline attention bias, whereas here all patients in the ABM condition were trained away from threat. These important differences could potentially explicate the deviation of the current results from these previous RCTs.

The overall reduction in symptoms across groups could reflect: (a) a simple time effect often observed in symptomatic patients showing spontaneous decrease in symptoms (McDonald, Mazzuca, & McCabe, 1983); (b) expectancy effects (Constantino, Visla, Coyne, & Boswell, 2018; Kazdin, 1979). This possibility, however, may be less likely because treatment expectancy did not correlate with symptom change. Finally, (c) the speedy (4 weeks) symptom reduction across groups may suggest that both ABM and ACT include active components, including a targeting of attention control mechanisms (Mogg, Waters, & Bradley, 2017; Pettit et al., 2020). This possibility could be tested directly in future studies by measuring attention control and applying non-active control conditions. Other factors that were comparable in both groups such as exposure to threatening faces or the attention received from the assistants and clinicians throughout the treatment could also be considered as contributors to symptomatic reduction.

Our exploratory moderator analyses indicate that for patients without comorbid depression and for patients with shorter time elapsing since their traumatic event, ACT outperformed ABM in reducing PTSD symptoms while comorbidity with anxiety disorders did not moderate treatment outcomes. It is conceivable that previous in-clinic studies of ABM/ACT were conducted among samples with lower rates of depression compared to the current sample (Badura-Brack et al., 2015). The current findings are also compatible with a recent RCT showing the moderating effect of the time elapsed since trauma in patients with PTSD (Segal et al., 2020). Future studies could test these specific moderators using *a-priori* and pre-registered analyses.

The results of the current study should be considered in light of some limitations. First, the hypothesized change in attentional patterns did not emerge, that is, threat-related attention bias did not decrease in the ABM group and ABV decreased in both groups (and not specifically in the ACT group), suggesting that the treatments had possibly failed to engage their intended cognitive targets. It is noteworthy that the psychometrics of the attention bias indices in the current study were poor. While the classic attention bias score typically shows poor internal consistency (Rodebaugh et al., 2016), the ABV index in the current study had poorer reliability compared to that observed in previous studies (e.g. Alon et al., 2019). This could be due to larger variability in the end-equipment used by the different patients combined with less control over other environmental factors compared to in-clinic settings. Such variability could be overcome in future studies by lending standardized end-equipment to patients for the study duration. Alternatively, future studies could invite patients to in-clinic measurements and perhaps could use additional attention bias indices such as trial-level bias score variability (Zvielli, Bernstein, & Koster, 2015) or other more reliable methodologies such as eye-tracking that could also disentangle specific attention sub-components such as engagement with and disengagement from threat (Lazarov et al., 2019*b*; Mekawi et al., 2020). Second, the present study did not include attention control measurement or indices of other cognitive mechanisms that may underlie the efficacy of both ACT and ABM. Future studies are encouraged to incorporate such potential common factors for ACT and ABM (Linetzky, Pettit, Silverman, Pine, & Bar-Haim, 2020). The fact that ABV reduced in the two conditions hints that both strengthened attentional control in the context of attentional competition between threat and neutral stimuli (Bardeen, Tull, Daniel, Evenden, & Stevens, 2016; Clarke et al., 2020). Third, even though the current study has shown supervised ACT/ABM to be a highly acceptable treatment, the current design did not permit direct examination of efficacy relative to remote non-supervised or in-clinic protocols. Future studies are encouraged to directly compare the efficacy of such protocols in a full factorial design of location (lab/home) and supervision (supervised/non-supervised).

To conclude, this is the first study to apply *supervised* remotely-delivered ABM/ACT protocols for patients with PTSD. The current study replicates medium-to-large clinical effects for dot-probe-based ACT/ABM protocols in reducing PTSD symptoms (Badura-Brack et al., 2015; Lazarov et al., 2019*a*), but failed to confirm an advantage of ACT over ABM. Notably, dropout rates appear to be considerably lower with ABM/ACT for PTSD (6.7%) relative to dropout in other first-line in-clinic treatments (Imel et al., 2013) and Internet-delivered CBT (Lewis et al., 2019). If future head-to-head RCTs between ACT/ABM and first-line treatments for PTSD would indicate similar efficacy, then new treatment options with potentially greater access, lower costs, and lower dropout would emerge.

## Supplementary Material

supp

## Figures and Tables

**Table 1.** Baseline demographic characteristics, clinical measures, and attentional indices in the ACT and ABM groups

Variable	ACT	ABM	Statistic	*p* Value
Gender			χ^2^ = 0	1
Men	*n* = 20 (66.7%)	*n* = 20 (66.7%)		
Women	*n* = 10 (33.3%)	*n* = 10 (33.3%)		
Marital status			χ^2^ = 3.841	0.279
Single	*n* = 14 (46.7%)	*n* = 12 (40%)		
Married	*n* = 9 (30%)	*n* = 14 (46.7%)		
Divorced	*n* = 7 (23.3%)	*n* = 3 (10%)		
Widowed	*n* = 0 (0%)	*n* = 1 (3.3%)		
Religiousness			χ^2^ = 5.33	0.149
Secular	*n* = 24 (80%)	*n* = 18 (60%)		
Traditional	*n* = 3 (10%)	*n* = 8 (26.7%)		
Religious	*n* = 3 (10%)	*n* = 2 (6.7%)		
Ultra-orthodox	*n* = 0 (0%)	*n* = 2 (6.7%)		
Age	*M* = 38.93 (s.d. = 12.194)	*M* = 40.43 (s.d. = 12.36)	*t*(58) = 0.473	0.638
Years of education	*M* = 14.13 (s.d. = 2.27)	*M* = 14.58 (s.d. = 4.16)	*t*(58) = 0.52	0.605
Number of children	*M* = 1.63 (s.d. = 1.84)	*M* = 1.89 (s.d. = 2.36)	*t*(58) = 0.648	0.648
Trauma type			χ^2^ = 6.67	0.464
MVA	*n* = 9 (30%)	*n* = 9 (30%)		
Combat	*n* = 3 (10%)	*n* = 8 (26.7%)		
Sexual assault	*n* = 6 (20%)	*n* = 4 (13.3%)		
Terror attack/war	*n* = 6 (20%)	*n* = 4 (13.3%)		
Violent assault	*n* = 3 (10%)	*n* = 3 (10%)		
Medical incidents	*n* = 3 (10%)	*n* = 2 (6.7%)		
Time elapsed since traumatic event	*M* = 9.96 (s.d. = 9.21)	*M* = 11.63 (s.d. = 12.50)	*t*(58) = 0.589	0.558
Baseline comorbidity				
Depressive episode	*n* = 17 (56.7%)	*n* = 17 (56.7%)	χ^2^ = 0	1
Dysthymia	*n* = 2 (6.7%)	*n* = 4 (13.3%)	χ^2^ = 0.74	0.389
Social phobia	*n* = 7 (23.3%)	*n* = 7 (23.3%)	χ^2^ = 0	1
GAD	*n* = 11 (36.7%)	*n* = 7 (23.3%)	χ^2^ = 1.27	0.26
Panic disorder	*n* = 14 (46.7%)	*n* = 7 (23.3%)	χ^2^ = 3.59	0.058
Agoraphobia	*n* = 6 (20%)	*n* = 2 (6.7%)	χ^2^ = 2.31	0.129
OCD	*n* = 1 (3.3%)	*n* = 0 (0%)	χ^2^ = 1.02	0.313
CAPS – total score	*M* = 29.2 (s.d. = 6.733)	*M* = 31.9 (s.d. = 7.667)	*t*(58) = 1.449	0.153
PCL-5	*M* = 51.50 (s.d. = 10.36)	*M* = 54.36 (s.d. = 9.84)	*t*(58) = 1.098	0.277
PHQ-9	*M* =17.033 (s.d. = 5.123)	*M* = 16.8 (s.d. = 4.874)	*t*(58) = 0.181	0.857
CGI-S	*M* = 4.333 (s.d. = 0.547)	*M* = 4.467 (s.d. = 0.629)	*t*(58) = 0.876	0.384
Treatment credibility	*M* = 6.078 (s.d. = 1.625)	*M* = 5.456 (s.d. = 1.905)	*t*(58) = 1.361	0.179
Treatment expectancy	*M* = 5.531 (s.d. = 1.658)	*M* = 5.278 (s.d. = 1.729)	*t*(58) = 0.579	0.565
Attention bias	*M* = 0.237 (s.d. = 19.28)	*M* = 4.16 (s.d. = 20.54)	*t*(57) = 0.757	0.452
ABV	*M* = 0.069 (s.d. = 0.024)	*M* = 0.083 (s.d. = 0.041)	*t*(57) = 1.608	0.113

*Note.* MVA, motorvehicle accident; GAD, generalized anxiety disorder; CAPS, Clinician-Administered PTSD Scale; PCL, PTSD Checklist; PHQ, Patient Health Questionnaire; CGI-S, Clinical Global Impression Severity scale; ABV, attention bias variability.

## Footnotes

1 Responding to a review comment from ClinicalTrials.gov (13 January 2020), we mistakenly omitted the CAPS-5 PTSD diagnosis measure from the study registration (9 March 2020). Here, we report both severity scores and PTSD diagnosis.

## References

[R1] Alon Y, Naim R, Pine DS, Bliese PD, & Bar-Haim Y (2019). Validity of attention bias variability indices for posttraumatic stress disorder research: Evidence from patient data. Journal of Traumatic Stress, 32(5), 791–798. 10.1002/jts.22443. 31461560 PMC7678410

[R2] American Psychiatric Association. (2013). Trauma- and Stressor-Related Disorders. In Diagnostic and statistical manual of mental disorders, fifth edition (pp. 265–290). Arlington, VA: American Psychiatric Association. 10.1176/appi.books.9780890425596.744053.

[R3] Armstrong T, & Olatunji BO (2012). Eye tracking of attention in the affective disorders: A meta-analytic review and synthesis. Clinical Psychology Review, 32, 704–723. 10.1016/j.cpr.2012.09.004. 23059623 PMC3556338

[R4] Badura-Brack AS, Naim R, Ryan TJ, Levy O, Abend R, Khanna MM, … Bar-Haim Y (2015). Effect of attention training on attention bias variability and PTSD symptoms: Randomized controlled trials in Israeli and U.S. Combat Veterans. American Journal of Psychiatry, 172(12), 1233–1241. 10.1176/appi.ajp.2015.14121578. 26206075 PMC6343502

[R5] Bardeen JR, Tull MT, Daniel TA, Evenden J, & Stevens EN (2016). A preliminary investigation of the time course of attention bias variability in posttraumatic stress disorder: The moderating role of attentional control. Behaviour Change, 33(2), 94–111. 10.1017/bec.2016.5.

[R6] Bennabi D, Vandel P, Papaxanthis C, Pozzo T, & Haffen E (2013). Psychomotor retardation in depression: A systematic review of diagnostic, pathophysiologic, and therapeutic implications. BioMed Research International, 2013(Article ID 158746). 10.1155/2013/158746. PMC3830759 24286073

[R7] Berk M, Ng F, Dodd S, Callaly T, Campbell S, Bernardo M, … Trauer T (2008). The validity of the CGI severity and improvement scales as measures of clinical effectiveness suitable for routine clinical use. Journal of Evaluation in Clinical Practice, 14(6), 979–983. 10.1111/j.1365-2753.2007.00921.x. 18462279

[R8] Blevins CA, Weathers FW, Davis MT, Witte TK, & Domino JL (2015). The posttraumatic stress disorder checklist for DSM-5 (PCL-5): Development and initial psychometric evaluation. Journal of Traumatic Stress, 28(6), 489–498. 10.1002/jts.22059. 26606250

[R9] Bull CN, Krout JA, Rathbone-McCuan E, & Shreffler MJ (2001). Access and issues of equity in remote/rural areas. Journal of Rural Health, 17(4), 356–359. 10.1111/j.1748-0361.2001.tb00288.x. 12071561

[R10] Clarke PJF, Marinovic W, Todd J, Basanovic J, Chen NTM, & Notebaert L (2020). What is attention bias variability? Examining the potential roles of attention control and response time variability in its relationship with anxiety. Behaviour Research and Therapy, 135, 103751. 10.1016/j.brat.2020.103751. 33070010

[R11] Constantino MJ, Visla A, Coyne AE, & Boswell JF (2018). A meta-analysis of the association between patients’ early treatment outcome expectation and their posttreatment outcomes. Psychotherapy, 55(4), 473–485. 10.1037/pst0000169. 30335459

[R12] De Raedt R, & Koster EHW (2010). Understanding vulnerability for depression from a cognitive neuroscience perspective: A reappraisal of attentional factors and a new conceptual framework. Cognitive, Affective and Behavioral Neuroscience, 10(1), 50–70. 10.3758/CABN.10.1.50. 20233955

[R13] Devilly GJ, & Borkovec TD (2000). Psychometric properties of the credibility/expectancy questionnaire. Journal of Behavior Therapy and Experimental Psychiatry, 31(2), 73–86. 10.1016/S0005-7916(00)00012-4. 11132119

[R14] Fani N, Tone EB, Phifer J, Norrholm SD, Bradley B, Ressler KJ, … Jovanovic T (2012). Attention bias toward threat is associated with exaggerated fear expression and impaired extinction in PTSD. Psychological Medicine, 42(3), 533–543. 10.1017/S0033291711001565. 21854700 PMC3690118

[R15] Faul F, Erdfelder E, Lang AG, & Buchner A (2007). G*Power 3: A flexible statistical power analysis program for the social, behavioral, and biomedical sciences. Behavior Research Methods, 39(2), 175–191. 10.3758/BF03193146. 17695343

[R16] Guy W (1976). CGI clinical global impressions. In ECDEU assessment manual for psychopharmacology revised (pp. 217–221). Rockville (Md): National Institute of Mental Health.

[R17] Hankin CS, Spiro A, Miller DR, & Kazis L (1999). Mental disorders and mental health treatment among U.S. Department of Veterans Affairs outpatients: The Veterans Health Study. American Journal of Psychiatry, 156(12), 1924–1930. 10.1176/ajp.156.12.1924. 10588406

[R18] Hayes AF, & Rockwood NJ (2017). Regression-based statistical mediation and moderation analysis in clinical research: Observations, recommendations, and implementation. Behaviour Research and Therapy, 98, 39–57. 10.1016/j.brat.2016.11.001. 27865431

[R19] Hoge CW, Castro CA, Messer SC, McGurk D, Cotting DI, & Koffman RL (2004). Combat duty in Iraq and Afghanistan, mental health problems and barriers to care. U.S. Army Medical Department Journal, 351, 7–17. 10.1056/NEJMoa040603. 20088060

[R20] Iacoviello BM, Wu G, Abend R, Murrough JW, Feder A, Fruchter E, … Charney DS (2014). Attention bias variability and symptoms of post-traumatic stress disorder. Journal of Traumatic Stress, 27, 232–239. 10.1002/jts. 24604631 PMC4617532

[R21] Imel ZE, Laska K, Jakupcak M, & Simpson TL (2013). Meta-analysis of dropout in treatments for posttraumatic stress disorder. Journal of Consulting and Clinical Psychology, 81(3), 394–404. 10.1037/a0031474. 23339535 PMC3893277

[R22] Kazdin AE (1979). Nonspecific treatment factors in psychotherapy outcome research. Journal of Consulting and Clinical Psychology, 47(5), 846–851. 10.1037/0022-006X.47.5.846. 512143

[R23] Kroenke K, Spitzer RL, & Williams JBWW (2001). The PHQ-9: Validity of a brief depression severity measure. Journal of General Internal Medicine, 16(9), 606–613. 10.1046/j.1525-1497.2001.016009606.x. 11556941 PMC1495268

[R24] Lazarov A, Suarez-Jimenez B, Abend R, Naim R, Shvil E, Helpman L, … Neria Y (2019a). Bias-contingent attention bias modification and attention control training in treatment of PTSD: A randomized control trial. Psychological Medicine, 49(14), 2432–2440. 10.1017/S0033291718003367. 30415648 PMC6520210

[R25] Lazarov A, Suarez-Jimenez B, Tamman A, Falzon L, Zhu X, Edmondson DE, & Neria Y (2019b). Attention to threat in posttraumatic stress disorder as indexed by eye-tracking indices: A systematic review. Psychological Medicine, 49, 705–726. 10.1017/S0033291718002313. 30178728 PMC6399079

[R26] Lewis C, Roberts NP, Simon N, Bethell A, & Bisson JI (2019). Internet-delivered cognitive behavioural therapy for post-traumatic stress disorder: Systematic review and meta-analysis. Acta Psychiatrica Scandinavica, 140(6), 508–521. 10.1111/acps.13079. 31359407

[R27] Liang KY, & Zeger SL (1986). Longitudinal data analysis using generalized linear models. Biometrika, 73(1), 13–22. 10.1093/biomet/73.1.13.

[R28] Linetzky M, Pergamin-Hight L, Pine DS, & Bar-Haim Y (2015). Quantitative evaluation of the clinical efficacy of attention bias modification treatment for anxiety disorders. Depression and Anxiety, 32(6), 383–391. 10.1002/da.22344. 25708991

[R29] Linetzky M, Pettit JW, Silverman WK, Pine DS, & Bar-Haim Y (2020). What drives symptom reduction in attention bias modification treatment? A randomized controlled experiment in clinically anxious youths. Clinical Psychological Science, 8(3), 506–518. 10.1177/2167702620902130.

[R30] Maguen S, Li Y, Madden E, Seal KH, Neylan TC, Patterson OV, … Shiner B (2019). Factors associated with completing evidence-based psychotherapy for PTSD among veterans in a national healthcare system. Psychiatry Research, 274, 112–128. 10.1016/j.psychres.2019.02.027. 30784780

[R31] McDermott LM, & Ebmeier KP (2009). A meta-analysis of depression severity and cognitive function. Journal of Affective Disorders, 119(1–3), 1–8. 10.1016/J.JAD.2009.04.022. 19428120

[R32] McDonald CJ, Mazzuca SA, & McCabe GP (1983). How much of the placebo ‘effect’ is really statistical regression? Statistics in Medicine, 2(4), 417–427. 10.1002/sim.4780020401. 6369471

[R33] Mekawi Y, Murphy L, Munoz A, Briscione M, Tone EB, Norrholm SD, … Powers A (2020). The role of negative affect in the association between attention bias to threat and posttraumatic stress: An eye-tracking study. Psychiatry Research, 284, 112674. 10.1016/J.PSYCHRES.2019.112674. 31831200 PMC7012707

[R34] Mogg K, Waters AM, & Bradley BP (2017). Attention bias modification (ABM): Review of effects of multisession ABM training on anxiety and threat-related attention in high-anxious individuals. Clinical Psychological Science, 5, 698–717. 10.1177/2167702617696359. 28752017 PMC5513441

[R35] Mogoaşe C, David D, & Koster EHW (2014). Clinical efficacy of attentional bias modification procedures: An updated meta-analysis. Journal of Clinical Psychology, 70(12), 1133–1157. 10.1002/JCLP.22081. 24652823

[R36] Morland LA, Wells SY, Glassman LH, Greene CJ, Hoffman JE, & Rosen CS (2020). Advances in PTSD treatment delivery: Review of findings and clinical considerations for the use of telehealth interventions for PTSD. Current Treatment Options in Psychiatry, 7, 221–241. 10.1007/s40501-020-00215-x. 32837831 PMC7261035

[R37] Naim R, Abend R, Wald I, Eldar S, Levi O, Fruchter E, … Bar-Haim Y (2015). Threat-related attention bias variability and posttraumatic stress. American Journal of Psychiatry, 172(12), 1242–1250. 10.1176/appi.ajp.2015.14121579. 26206076 PMC6335584

[R38] Niles AN, Woolley JD, Tripp P, Pesquita A, Vinogradov S, Neylan TC, & O’Donovan A (2020). Randomized controlled trial testing mobile-based attention-bias modification for posttraumatic stress using personalized word stimuli. Clinical Psychological Science, 8(4), 756–772. 10.1177/2167702620902119. 34414018 PMC8373050

[R39] Norris FH, Murphy AD, Baker CK, & Perilla JL (2004). Postdisaster PTSD over four waves of a panel study of Mexico’s 1999 flood. Journal of Traumatic Stress, 17(4), 283–292. 10.1023/B:JOTS.0000038476.87634.9b. 15462535

[R40] Pettit JW, Bechor M, Rey Y, Vasey MW, Abend R, Pine DS, … Silverman WK (2020). A randomized controlled trial of attention bias modification treatment in youth with treatment-resistant anxiety disorders. Journal of the American Academy of Child and Adolescent Psychiatry, 59(1), 157–165. 10.1016/j.jaac.2019.02.018. 30877049 PMC6744353

[R41] Rodebaugh TL, Scullin RB, Langer JK, Dixon DJ, Huppert JD, Bernstein A, … Lenze EJ (2016). Unreliability as a threat to understanding psychopathology: The cautionary tale of attentional bias. Journal of Abnormal Psychology, 125(6), 840–851. 10.1037/abn0000184. 27322741 PMC4980228

[R42] Rotnitzky A, & Jewell NP (1990). Hypothesis testing of regression parameters in semiparametric generalized linear models for cluster correlated data. Biometrika, 77(3), 485–497. 10.1093/biomet/77.3.485.

[R43] Schoorl M, Putman P, & van der Does W (2013). Attentional bias modification in posttraumatic stress disorder: A randomized controlled trial. Psychotherapy and Psychosomatics, 82(2), 99–105. 10.1159/000341920. 23295710

[R44] Segal A, Pine DS, & Bar-Haim Y (2020). Personalized attention control therapy for PTSD: Effectiveness and moderators of outcome in a randomized controlled trial. Psychological Medicine, 1–11. 10.1017/S0033291720004304. 33231534

[R45] Sheehan DV, Lecrubier Y, Sheehan KH, Amorim P, Janavs J, Weiller E, … Dunbar GC (1998). The Mini-International Neuropsychiatric Interview (M.I.N.I.): The development and validation of a structured diagnostic psychiatric interview for DSM-IV and ICD-10. The Journal of Clinical Psychiatry, 59(Suppl 20), 22–33. Retrieved from http://www.ncbi.nlm.nih.gov/pubmed/9881538. 9881538

[R46] Spinhoven P, Penninx BW, van Hemert AM, de Rooij M, & Elzinga BM (2014). Comorbidity of PTSD in anxiety and depressive disorders: Prevalence and shared risk factors. Child Abuse and Neglect, 38(8), 1320–1330. 10.1016/j.chiabu.2014.01.017. 24629482

[R47] Straud CL, Siev J, Messer S, & Zalta AK (2019). Examining military population and trauma type as moderators of treatment outcome for first-line psychotherapies for PTSD: A meta-analysis. Journal of Anxiety Disorders, 67, 102133. 10.1016/j.janxdis.2019.102133. 31472332 PMC6739153

[R48] Weathers FW, Bovin MJ, Lee DJ, Sloan DM, Schnurr PP, Kaloupek DG, … Marx BP (2018). The clinician-administered PTSD scale for DSM-5 (CAPS-5): Development and initial psychometric evaluation in military veterans. Psychological Assessment, 30(3), 383–395. 10.1037/pas0000486. 28493729 PMC5805662

[R49] Zeger SL, Liang K-Y, & Albert PS (1988). Models for longitudinal data: A generalized estimating equation approach. Biometrics, 44(4), 1049. 10.2307/2531734. 3233245

[R50] Zvielli A, Bernstein A, & Koster EHW (2015). Temporal dynamics of attentional bias. Clinical Psychological Science, 3(5), 772–788. 10.1177/2167702614551572.

