# Supplementary material for: A randomized controlled trial of supervised remotely-delivered attention bias modification for posttraumatic stress disorder
Source: Psychol Med. 2022 Feb 8;53(8):3601–10. doi: 10.1017/S003329172200023X (PMC10248784; doi:10.1017/S003329172200023X)
Supplement: Supplementary file 1 [file S003329172200023Xsup001.docx]

| **Table S1.** Issues warranting improvement and merits of the current protocol derived from patients' free feedback given post-treatment. | |
| --- | --- |
| **Issues warranting improvement** | **N (%) of patients relating to this issue** |
| Treatment’s rationale was not clear enough and should be better explained | 20 (33.33%) |
| Treatment’s task is too monotonous/not challenging enough | 10 (16.67%) |
| Scarcity of interactions with clinicians and research assistants | 7 (11.67%) |
| Reading instructions at the beginning of each session was unnecessary | 6 (10%) |
| Exposure to facial stimuli was unpleasant | 4 (6.67%) |
| Using the internet was difficult | 2 (3.33%) |
| **Merits of the novel protocol** | **N (%) of patients relating to this issue** |
| Remote delivery was convenient and saved time on the roads | 23 (38.33%) |
| The interaction with study’s personnel was pleasant | 20 (33.33%) |
| Research personnel was professional | 4 (6.67%) |
| Reminders and interactions with research personnel aided in treatment adherence | 3 (5%) |
| Sessions are short and thus save time | 2 (3.33%) |
